# Multiple Sclerosis and Oligodendroglioma: An Exceptional Association

**DOI:** 10.1155/2014/546817

**Published:** 2014-08-07

**Authors:** Ana Teresa Carvalho, Paulo Linhares, Lígia Castro, Maria José Sá

**Affiliations:** ^1^Neurology Department, Centro Hospitalar Vila Nova Gaia/Espinho, 4434-502 Vila Nova de Gaia, Portugal; ^2^MS Clinic, Neurology Department, Centro Hospitalar São João, 4440-563 Porto, Portugal; ^3^Neurosurgery Department, Centro Hospitalar São João, 4440-563 Porto, Portugal; ^4^Faculty of Medicine of Porto University, 4200-319 Porto, Portugal; ^5^Pathological Anatomy Department, Centro Hospitalar São João, 4440-563 Porto, Portugal; ^6^Faculty of Health Sciences, Fernando Pessoa University, 4249-004 Porto, Portugal

## Abstract

The cooccurrence of multiple sclerosis (MS) and oligodendroglioma is very rare. We present a 43-year-old male patient with the diagnosis of MS lasting for 14 years who developed seizures and right hemiparesis; cerebral MRI revealed an already known extensive lesion, previously misdiagnosed as tumefactive demyelinating lesion. Cerebral biopsy leads to oligodendroglioma diagnosis, successfully treated with radiotherapy. The diagnosis of a brain tumor in a MS patient is challenging. The atypical clinical and radiological features are the key for accurate diagnosis. In such cases, a brain tumor has to be kept in mind no matter how rare this association is.

## 1. Introduction

The global risk of cancer among patients with multiple sclerosis (MS) seems to be lower than in general population with a reported incidence of 1.75% [1]. This lower incidence of tumors in MS patients has been explained by several factors, including greater clinical surveillance and health care, some immunologic characteristics of MS disease activity that improve antitumor surveillance, and underdiagnosis, since new neurological symptoms in MS patients are easily attributed to a new relapse, which usually implies immediate steroid treatment. In addition, tumors may be misdiagnosed on MRI by attributing the respective lesion to a tumefactive MS form. The cooccurrence of multiple sclerosis (MS) and pure oligodendroglioma is even rarer, with only 7 cases reported in the literature since 1967 [2–7] (Table 1).

Oligodendroglioma accounts for approximately 2.5% of all primary brain tumours and 5-6% of all gliomas [8]. Males appear to be affected slightly more frequently than females, with a ratio of 1.1 : 1 [8], as oligodendrogliomas typically develop in the 5th decade of life and usually involve the frontal or (less commonly) the temporal lobe. Seizures are the most frequent clinical presentation. In this paper, we describe a patient with MS who developed a pure oligodendroglioma.

## 2. Case Report

**Figure 1:**
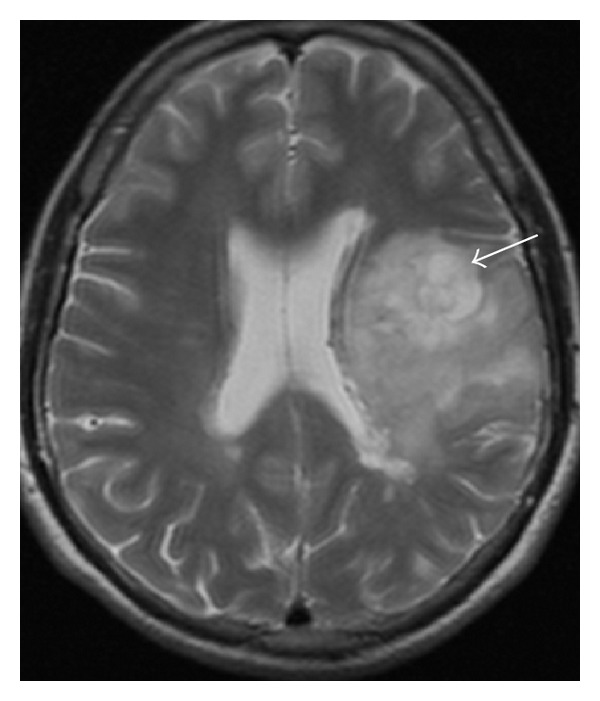
Cerebral MRI (2003): large left frontal lobe lesion (arrow) suggesting a tumefactive demyelinating lesion.

**Figure 2:**
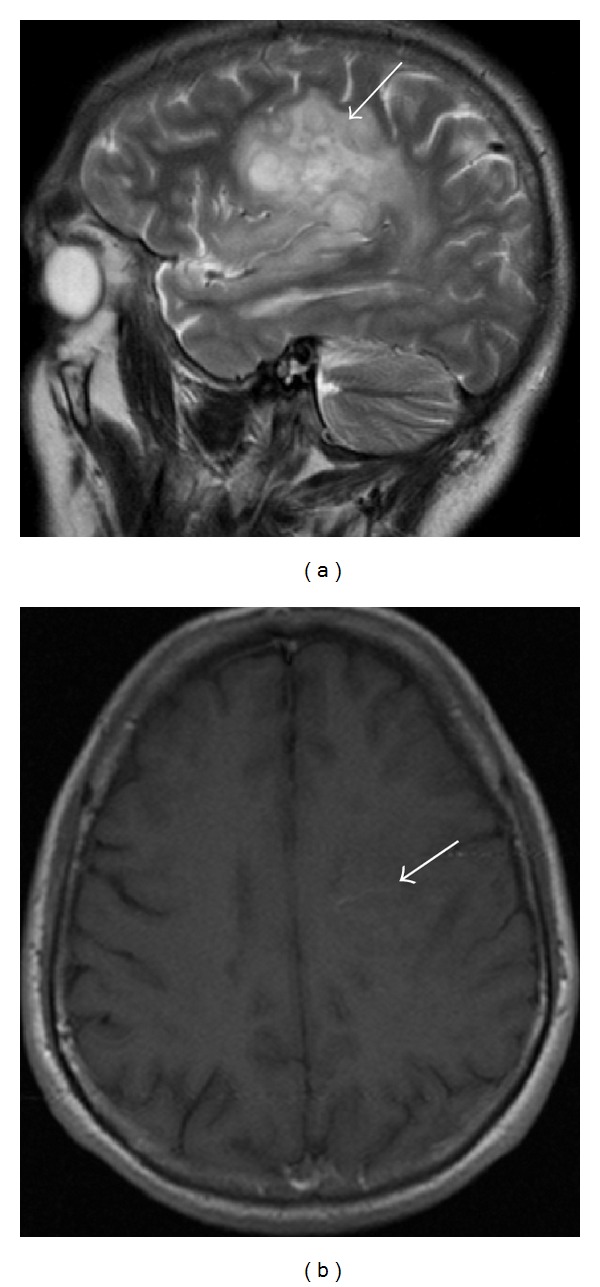
Cerebral MRI (2008): (a) large lesion in the left frontal lobe (large arrow). (b) Subtle contrast-enhancement (small arrow) and mass effect, suggesting an infiltrative lesion from glial series.

**Figure 3:**
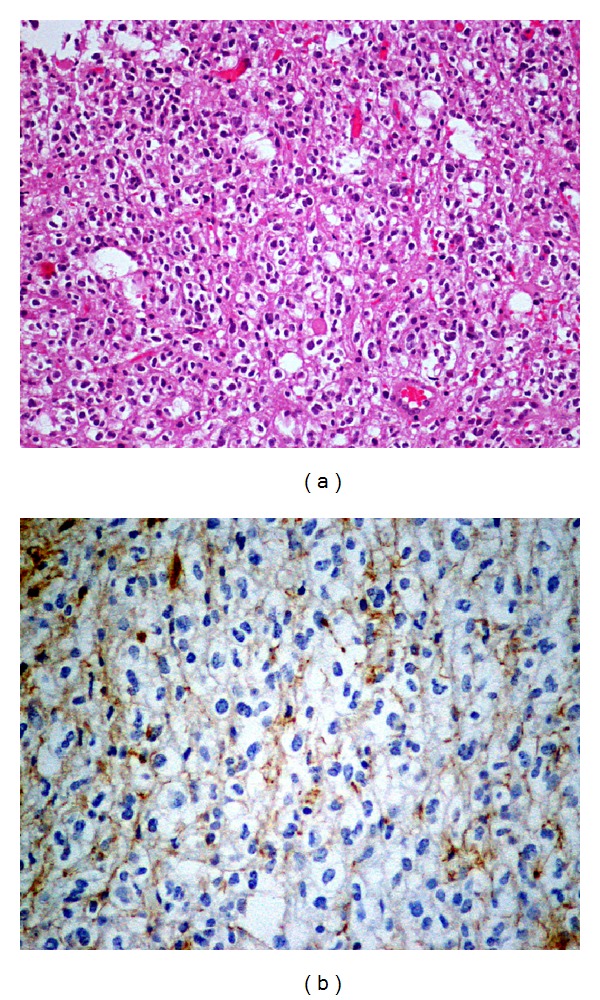
Histological examination. (a) Hematoxylin/Eosin coloration, with ×20 magnification, reveals moderately cellular neoplasm, consisting of cells with round nuclei and perinuclear light halo. (b) GFAP coloration, with ×40 magnification, reveals focal immunoreactivity of neoplastic cells (GFAP positive, CD68 negative cells).

A 43-year-old male patient is followed in our MS clinic since 1994 with the diagnosis of clinical definite relapsing-remitting MS. The clinical presentation was a grade 4 paraparesis with no other symptoms; the initial MRI showed typical lesions for MS and cerebrospinal fluid analysis revealed positive oligoclonal bands. Interferon beta 1-b was started as treatment in 2003 with clinical efficacy and the patient had a full recovery of the paraparesis. In 2003 a routine brain MRI revealed a new extensive subcortical and deeper white matter lesion localized in the left frontal lobe, which is noncontrast-enhancing, suggesting a tumefactive demyelinating lesion (Figure 1). There were no clinical changes and imagiological characteristics of this lesion remained unchanged in consecutive MRIs. In 2006, despite the absence of new symptoms or relapses, a poor therapeutic adherence was documented and switch to glatiramer acetate (GA) was performed. In 2008, the patient presented with partial complex and generalized seizures in association with progressive right hemiparesis. A new cerebral MRI (Figure 2) revealed again the large left frontal lesion, but now with space-occupying characteristics, subtle contrast-enhancement, and mass effect, suggesting an infiltrative lesion of glial series. On this basis, a presumptive diagnosis of low grade glioma was made and a cerebral biopsy was performed. Histological examination (Figure 3) revealed cells with clear cytoplasm, round nuclei, and granular chromatin; glial fibrillary acidic protein (GFAP) immunopositivity evidenced neoplastic cells expression; proliferation index was less than 5%. These features were found to be diagnostic of a World Health Organization grade 2 oligodendroglioma. GA was interrupted and patient underwent treatment with conventional fractioned radiotherapy with 30 fractions of 2 Gy to total dose of 60 Gy. Seizures were controlled with valproic acid 1000 mg per day and levetiracetam 1500 mg per day. At 3-year follow-up, sequential MRI revealed both demyelinating and neoplastic stable lesions. After 3 years of follow-up, the patient presents minor right hemiparesis and dysarthria.

## 3. Discussion

The diagnosis of a brain tumor in a MS patient is challenging, due to several reasons that include the fact that new neurological symptoms in MS patients are easily attributed to a relapse of the disease and the MRI lesions, even if suspicious, are commonly diagnosed or confused as a tumefactive form of MS. Also the cooccurrence of two neurological diseases in the same patient is uncommon, particularly oligodendroglioma and MS. In fact, several years before the tumor suspect, routine MRI revealed an atypical new extensive lesion, similar to others reported cases [5–7] (Table 1). Although he was asymptomatic at that time, maybe images were undervalued because he had already MS diagnosis. Indeed, in a previously healthy patient, a neoplastic etiology would probably be easier; however in a patient with clearly established MS a tumefactive form of demyelinating disease is the commonest diagnosis, in as much as the cooccurrence of MS and brain tumors is unusual and also the occurrence of a brain tumor in these patients is less than in general population. Only when our patient presented an atypical MS symptom—a seizure—a suspect of another diagnosis was considered, as in previously reported cases [6, 7] (Table 1). In effect, seizures, as well as other symptoms atypical for MS, such as headache, aphasia, agnosia, and visual fields defects, are recognized as clinical red flag for MS [9] implying the search of alternate diagnosis. However, those symptoms lack specificity since they may occur in brain neoplasm and tumefactive MS—the main differential diagnosis—and even in classical MS with cortical lesions.

Radiological red flags for MS, that is, atypical MRI features, include size of >2 cm, mass effect, perilesional oedema, and/or atypical enhancement (such as complete ring or heterogeneity), but they may occur in both tumefactive demyelinating lesions (TDL) and malignancies. In comparison with tumor, mass effect and oedema in MS are proportionally minor relative to plaque size [9]. The use of additional techniques may be helpful in differentiating lesions' nature. The utility of magnetization transfer has been not systematically explored, but apparently both neoplastic processes and tumefactive demyelinating lesions can show a similar decline in magnetization transfer values. Perfusion-weighted MRI (PWI) does not look extremely helpful in differentiating tumefactive lesions from tumors, since necrotic neoplasms may display a similar increase in diffusion coefficients centrally within the lesion. Diffusion tensor imaging (DTI) is said to be helpful in differentiating TDL from high-grade gliomas by using visual inspection and quantitative analysis: TDL have a significantly higher incidence of intralesional hyperintensities on fractional anisotropy (FA) maps but a lower incidence of a perilesional hyperintense FA rim, compared with those of high-grade gliomas on visual inspection. TDL had significantly higher FA and lower mean diffusivity values in the peripheral enhancing portions of the lesions compared with those of high-grade gliomas. In perilesional edema, FA values were significantly higher in high-grade gliomas. Butteriss and colleagues [10] proposed the use of serial proton MR spectroscopy (1H MRS) to noninvasively differentiate glioma from tumefactive plaque in a MS patient, concluding that persistently elevated choline was more suggestive of neoplasm, rather than an inflammatory process (although elevated choline levels were described in chronic MS plaques). Thallium-201 single-photon emission computed tomography (SPECT) and 18-FDG positron emission tomography (PET) may be applied for an accurate differential diagnosis of a brain tumor. So, cerebral biopsy is the accurate way to confirm the diagnosis.

It is worth noting that oligodendroglioma is a tumor derived from the glial cell involved in myelin production and hence in the MS physiopathology. In this sense, a causal association between oligodendroglioma and MS has been already proposed [11]. Some authors suggested that this tumor develops from neoplastic transformation of reactive glial cells in areas of established demyelination [11]. In fact, oligodendrocyte was thought to be a cell without regeneration proprieties, but some experimental studies demonstrate that it is able to divide itself in adult animals (mouse) and, when it is destroyed, other mature oligodendrocytes can replace it [7]. Remyelination requires oligodendrocytes to undergo at least one cellular division [12]. So, demyelinating disease may cause oligodendrocytes to reenter the mitotic cycle which may induce a neoplastic transformation in response to injury [13]. Also, a gradual histological transition from areas with reactive gliosis related to demyelination to overt glial tumors on autopsy studies was demonstrated.

The cause-effect hypothesis is also based on the temporal sequence: tumor usually occurs many years (average of 15 years) after MS onset. On the other hand, these tumors tend to be multicentric. From gliomas occurring in MS patients, nearly 30 percent are multicentric or diffusely infiltrative, against 2.5 to 5 percent of gliomas unassociated with MS [14]. the possibility that shared genetic factors might underlie susceptibility to both conditions was also considered. Studies in regions with high prevalence of MS (Scandinavia and northern Europe) involving large series of patients with MS and intracranial neoplasms suggest a higher than expected incidence of oligodendroglioma [15].

The role of long-term exposure of MS patients to first line immunomodulatory drugs (interferon-beta and GA) remains controversial. There is one report of intracranial neoplasm (medulloblastoma) in a MS patient taking GA [16]. The established association between GA treatment and neurogenesis leads the authors to consider a possible role of GA as an inducer of abnormal neurogenesis is MS patients.

However, as the reported cases are so exceptional, the association between oligodendroglioma and MS could be explained merely by coincidence.

## 4. Conclusions

As far as we know from the literature review, this is the 8th reported case of pure oligodendroglioma and MS cooccurrence. The atypical clinical and radiological features in MS patients are the key for accurate diagnosis. If a pseudotumoral form of MS is diagnosed, a careful clinical and radiological follow-up is required. In such cases, a brain tumor has to be kept in mind no matter how rare this association is. The lesion biopsy is the only accurate method for definitive diagnosis and should be used as soon as new atypical lesions appear.

## Figures and Tables

**Table 1:** Reported cases of MS and pure oligodendroglioma cooccurrence.

Author		Age at MS onset	Age at tumor diagnosis	MS course	Tumor clinical presentation	MRI main features (other than typical MS findings)	Histopathology
Khan et al. [5]		43	51	RRMS	No symptoms (suspicion based on routine MRI)	3 cm enhancing lesion involving both white and gray matter in the right parietal lobe∗	Monomorphic neoplasm composed of oligodendrocytes, GFAP positive, and CD68 negative

Green et al. [6]	Case 1	34	50	RRMS	No symptoms (suspicion based on routine MRI)	Nonenhancing mass lesion involving white matter in the right temporoparietal area, with some sulcal effacement of the overlying cortex∗	Microcystic low grade oligodendroglioma
	Case 2	44?	44?	N/A	Seizures and monocular inferior left scotoma	Right temporal lobe lesion with some mass effect∗	Grade 2 oligodendroglioma

Giordana et al. [3]		34	42	PPMS	N/A	Bilateral frontal lesion, involving anterior *corpus callosum *	Unclear grade oligodendroglioma, not contiguous with MS lesions

Rao et al. [4]		N/A	65	N/A	N/A	Diffuse lesion (right > left) across *corpus callosum *	Oligodendroglioma with spotty calcification, ring lesions, contiguous with MS plaques

Barnard and Jellinek [2]		28	43	RR/SC MS	N/A	Mass lesion at the right temporal lobe	“Polymorphic” oligodendroglioma with mitotic figures and central necrosis

de la Lama et al. [7]		26	37	RRMS	Seizures	Large subcortical lesion at the right frontal lobe, poorly enhancing, causing mass effect and midline shifting to the left∗	Tumoural proliferation with homogeneous nuclei and clear cytoplasms, not contiguous with MS plaques—grade C oligodendroglioma (Smith classification)

∗Enlargement from previously known lesion in routine MRI.

N/A: not available; RRMS: relapsing-remitting multiple sclerosis; SC: secondary progressive.
